# Increased Provision of Bioavailable Mg through Vegetables Could Significantly Reduce the Growing Health and Economic Burden Caused by Mg Malnutrition

**DOI:** 10.3390/foods10112513

**Published:** 2021-10-20

**Authors:** Dunyi Liu, Ming Lu, Prakash Lakshmanan, Ziyi Hu, Xinping Chen

**Affiliations:** 1 Key Laboratory of Efficient Utilization of Soil and Fertilizer Resources, College of Resources and Environment, Southwest University, Chongqing 400715, China; liudy1989@swu.edu.cn (D.L.); xiaomeinv0324@163.com (M.L.); huziyi@email.swu.edu.cn (Z.H.); 2 Interdisciplinary Research Center for Agriculture Green Development in Yangtze River Basin, Southwest University, Chongqing 400715, China; plakshmanan2018@outlook.com; 3 Sugarcane Research Institute, Guangxi Academy of Agricultural Sciences, Nanning 530007, China; 4 Queensland Alliance for Agriculture and Food Innovation, University of Queensland, St. Lucia, QLD 4067, Australia

**Keywords:** vegetable Mg, health burden, economic cost, Mg biofortification

## Abstract

Magnesium (Mg) is an essential mineral nutrient for human health and its deficiency associated with many diseases, including stroke, heart failure, and type 2 diabetes. Vegetables are an important source of dietary Mg for humans. In this study, we quantified vegetable Mg content by a global meat analysis, analyzed human health, and economic impact caused by Mg deficiency. Results revealed that vegetable Mg content showed a large variation with an average value of 19.3 mg 100 g^−1^ FW. Variation in per capita vegetable-Mg supply in different continents is largely ascribed to continental difference in the amount and the type of vegetables produced. The health and economic loss attributed to Mg deficiency are estimated to be 1.91 million disability-adjusted life years (DALYs) and 15.8 billion dollars (0.14% of GDP), respectively. A scenario analysis indicated that the increasing vegetable production (increased by 8.9% and 20.7% relative to 2017 in 2030 and 2050) and vegetable Mg content (increased by 22% through biofortification) could significantly reduce DALYs (1.24 million years) and economic burden (0.09% of GDP). This study could guide a major re-balance of production practices, species cultivated, and Mg biofortification to provide sufficient vegetable Mg for better human Mg nutrition.

## 1. Introduction

Magnesium (Mg) is the fourth most abundant mineral in human body and is required by all living cells [1]. Mg is a co-factor for more than 300 human metabolic reactions, it is also indispensable in energy production, synthesis of RNA and DNA, and bone development [2,3]. Mg deficiency, defined as the serum Mg content <0.75 mmol/L [4], causes many health issues including cardiovascular diseases [5], type 2 diabetes [6], muscular disorders [7], and osteoporosis [8]. To prevent deficiency, the recommended daily allowance (RDA) of Mg for adults is 310–420 mg/day depending on the age and gender [9]. A meta-analysis revealed that increasing dietary Mg intake by 100 mg/day in people with Mg deficiency could decrease the risk of stroke, heart failure and type 2 diabetes by 7%, 22% and 19%, respectively [10]. Globally, a large number of people are Mg deficient, especially those consuming excessive amounts of alcohol, coffee, tea, and highly processed foods that are inherently low in Mg content [11,12]. Dietary surveys showed that about 75 percent of Americans do not meet the RDA for Mg [13]. Though Mg supplements are increasingly used in developed countries (around 7% in general population and 25% among athletes) [14], preventing wide-spread Mg deficiency through food is now considered as an important and effective strategy to manage this issue [9]. Factors influencing Mg bioavailability in plant-based foods include nature of the food matrix and Mg form in the food, interactions between Mg and inorganic/organic components of food (e.g., potassium, phytate, dietary fibres, proteins), pre-treatment of food (e.g., heat treatment) [15]. In nature, vegetables are a major source of Mg with the green leafy vegetables being a particularly rich source of this important nutrient [16]. Vegetables, fruits, nuts and grains provide for about 45% of human dietary Mg requirement [17]. However, the Mg content in cereals showed a steady, significant decline during the past several decades, possibly due to narrowing of genetic diversity among commercially cultivated genotypes due to increased focus on grain yield and processing quality [18]. The problem is further aggravated by the heavy processing of cereals and legumes, which causes substantial loss of Mg. For example, when wheat is milled into wheat flour, there is an approximate 80% loss of Mg [19]. Given this scenario, vegetables present an effective and widely applicable delivery route to increase dietary Mg intake, and is likely to play an increasingly important role in managing human Mg nutrition.

Over the past 50 years, global vegetable production has increased tremendously, and its consumption is steadily increasing [20]. But vegetable production remains unbalanced in different regions of the world due to population growth, economic disparity, eating habits and other factors [21]. Globally vegetable production is primarily determined by the culturally ingrained preferences shown by the local population. Only 5% of the vegetables grown are traded internationally [22]. Various studies showed large regional differences in Mg supply by vegetables. In addition, Mg content varies greatly among different vegetable varieties. For instance, in Australia, vegetable Mg content ranges from 2.0–34 mg 100 g^−1^ fresh weight (FW), with pepper and lettuce showing the largest variation [23]. A significant difference in Mg content was reported among inbred and hybrid entries of broccoli heads, and it is strongly affected by environment [24]. Furthermore, the local vegetable diversity and crop production constraints and practices also have a great impact on plant nutrition and human health. A combination of these factors may underpin the significant regional variation in Mg supply from vegetables, which needs to be quantified in order to find ways of improving human Mg nutrition and public health.

Mg biofortification through breeding and/or agronomic practices is a viable option for improving vegetable Mg content [18,25]. The agronomic approach, which generally relies on formulated fertilizers, is evolving as an effective approach for increasing plant mineral content rather quickly. The effectiveness of this approach has been demonstrated with produces biofortified with calcium (Ca), iodine (I), zinc (Zn), selenium (Se), iron (Fe), copper (Cu), and silicon (Si) that increased their dietary intake with an attendant reduction in deficiency [25,26,27,28]. A similar approach of Mg biofortification of Italian ryegrass is being pursued for grazing ruminants [29,30].

The disability-adjusted life years (DALYs) approach is commonly used to assess the health burden of a disease or disorder. DALYs are the sum of the years of life lost (YLLs) and the years lived with a disability (YLDs) due to the disease or health condition in a population [31]. It can also be used to assess nutrient deficiency-induced health burden [32]. The DALYs saved, means the DALYs reduced by effective measures (e.g., biofortification), could be used to quantify the impact of these measures on human health [28,32]. Though DALYs are key considerations for estimating the burden of diseases in population, it will not capture the complete adverse impact of diseases on human life. In particular, in this context the economic consequences of poor health can be substantial [33]. Increasingly research efforts are being directed to quantify the economic costs of diseases [34,35]. However, such studies on quantifying health and economic burden caused by Mg deficiency and their alleviation by biofortification are limited.

The objectives of this study were (1) to quantify Mg contents in common vegetables (based on 2412 independent data points reported in 235 published references of 12 popular vegetables) and its variation among continents; (2) to estimate the health (DALYs) and economic cost caused by Mg deficiency using China as a case study; and (3) to determine the potential health and economic impact of Mg-biofortified vegetables.

## 2. Materials and Methods

### 2.1. Magnesium Content in Major Vegetables

According to the production quantities, tomato, onion, spinach, garlic, cauliflower, cabbage, eggplant, lettuce, cucumber, pepper, turnips, vegetable fresh nes. They account for ~88% of total global vegetable production (Table S1). Based on FAO crop database [20], vegetables fresh nes include inter alia: bamboo shoots, beets, chards, capers, cardoons, celery, chervil, cress, fennel, horseradish, sweet marjoram, oyster plant, parsley, parsnips, radish, rhubarb, rutabagas, swedes, savory, scorzonera, sorrel, soybean sprouts, tarragon and watercress. A survey of peer-reviewed papers published from January 1 1940 to 1 January 2018 was conducted using ISI-Web of Science (Thomson Reuters, New York, NY, USA), Google Scholar (Google Inc., Mountain View, CA, USA), and the China Knowledge Resource Integrated database (CNKI) to establish the edible-part vegetable Mg contents database for different vegetable species. To minimize bias, we used the following criteria to select studies based on the procedure of meta-analysis [36]: (i) the Mg contents were from edible parts of the vegetable and (ii) the measured stage for Mg contents was at maturity. Based on the literature survey, we assembled 235 published references (containing 2412 independent data points) for Mg contents for different vegetable species (Supplementary Materials, Extended reference list). Since Mg contents in dry weight were presented in the literature, we unified the results into fresh weight according to the moisture content (Table S1) of each vegetable.

Mg content of each vegetable species reported is the median value obtained from the meta-analysis. The vegetable Mg production in the world or in each continent during the last 50 years was defined as vegetable Mg content multiplied by the vegetable production in the corresponding continent. The vegetable production during the last 50 years (1968–2017) was derived from the FAO crop database. The vegetable Mg content was defined as the weighted average of Mg content value of all vegetable species, taking the total production amount of each vegetable as its weight. Vegetable Mg supply per day^−1^ capita^−1^ in the world and in each continent was defined as vegetable Mg production divided by total population and by days. The continents/regions used in this study were Asia, Europe, North America, South America, Africa, and Oceania. Vegetables were classified into 3 groups based on their Mg content. High Mg vegetables: those containing >30 mg Mg 100 g^−1^ FW (include spinach and garlic); Medium Mg vegetables: those containing Mg content from 20 to 30 mg 100 g^−1^ FW (include cauliflower, vegetable fresh nes, and cabbage); Low Mg vegetables: those containing Mg content <20 mg 100 g^−1^ FW (include eggplant, lettuce, onions, cucumber, pepper, tomato, turnips and others).

### 2.2. Health and Economic Burden Attributed to Mg Deficiency

Functional outcomes attributed to Mg deficiency and adverse health outcomes, supported by robust and conclusive data, were used for analyses. Thus, we selected three major health conditions (stroke, heart failure and type 2 diabetes) and took China as an example to calculate the health and economic burden attributed to Mg deficiency. The DALYs equation was used to calculate the health burden of Mg deficiency, which was expressed as the sum of YLD and YLL from disease. The YLD and YLL are based on the following formula [32]:

(1) $$\;\mathrm{YLD}=\underset{j}{\sum }TjIijDj\left(\frac{1-e^{-rdij}}{r}\right)$$

(2) $$\;\mathrm{YLL}=\underset{j}{\sum }TjMj\left(\frac{1-e^{-rLj}}{r}\right)$$

*TjIijDj*, the number of disabilities due to Mg deficiency, calculated by the total number of people in the target group *j* multiplied by incidence rate of functional outcome *i* in target group *j* multiplied by disability weight for functional outcome *i* in target group *j*; *dij*, duration of functional outcome *i* in target group *j*; *r*, discount rate for future life years, 3% is applied [37]; *TjMj*, the number of deaths due to Mg deficiency, calculated by the total number of people in the target group *j* multiplied by the mortality rate associated with Mg deficiency in target group *j*; *Lj*, average remaining life expectancy for target group *j*.

Based on a human resources approach from a social perspective, the economic burden was estimated, which included direct costs (e.g., cost of healthcare service) and indirect costs (e.g., loss of productivity). The direct costs of outcomes attributed to Mg deficiency was derived from literature, and the indirect costs was calculated by DALYs lost multiplied by GDP per capita.

### 2.3. Scenario Analysis

Based on the following formula, we estimated the Mg intake from vegetables, the number of cases (including deaths and disabilities) due to Mg deficiency, DALYs lost, DALYs saved, economic burden attributed to Mg deficiency and its percentage to GDP in the year 2017 (at present), 2030 and 2050 (in the future).

To evaluate the improvement of vegetable Mg content through biofortification, the improvement ratio was calculated by:

Ratio *= BC*/*CC* × 100 (3)

where *BC* is the vegetable Mg content with biofortification; *CC*, current vegetable Mg content. These values were deceived from the meta-analysis on vegetable Mg contents.

Mg intake from vegetables was calculated by:

Mg_intake_ = Mg_supply_ × (1 − Wasted%) (4)

where Mg_intake_, Mg intake from vegetables; Mg_supply_, vegetable Mg supply per day^−1^ capita^−1^; Wasted%, percentage waste of vegetables during agricultural production, postharvest handling and storage, processing and packaging, distribution and consumption, which was estimated to be 43% in China [38].

In order to determine the degree of health impact with Mg biofortification and increased vegetable production, DALYs saved was calculated [32,39]:

(5) $$\mathrm{DALYs}\;\mathrm{saved}=\frac{ln\left(\frac{BI}{CI}\right)-\left(\frac{BI-CI}{RDA}\right)}{ln\left(\frac{RDA}{CI}\right)-\left(\frac{RDA-CI}{RDA}\right)}\times Ct\times \mathrm{DALYs}\;\mathrm{lost}$$

where *CI* is the current Mg intake and *BI* is the Mg intake with biofortification; *RDA*, the recommended daily allowance of Mg, which was set at 400 mg day^−1^ in the current study; *Ct*, the biofortification technology adoption rate of 60% is applied, which represents an optimistic scenario [32].

4 scenarios were set: Business as usual, which represents vegetable production in 2030 and 2050 following the historical trend while the current vegetable Mg content is maintained (B2030 and B2050); Scenario, with improved vegetable Mg content based on B2030 and B2050 through biofortification (S2030 and S2050).

### 2.4. Statistical Analysis

The primary data were processed and analysed using Microsoft Office Excel 2013 and Sigmaplot (Ver. 12.0 for windows, SYSTAT Inc., San Jose, CA, USA). The vegetable Mg contents were reported with 95% confidence intervals (CIs). According to Kolmogorov–Smirnov analysis (SPSS Inc., Chicago, IL, USA), we found a normal distribution of improvement ratio of vegetable Mg content by biofortification, suggesting the data is suitable for meta-analysis.

## 3. Results

### 3.1. Large Variation in Vegetable Mg Content and Imbalanced Vegetable Production Exist Globally

**Figure 1:**
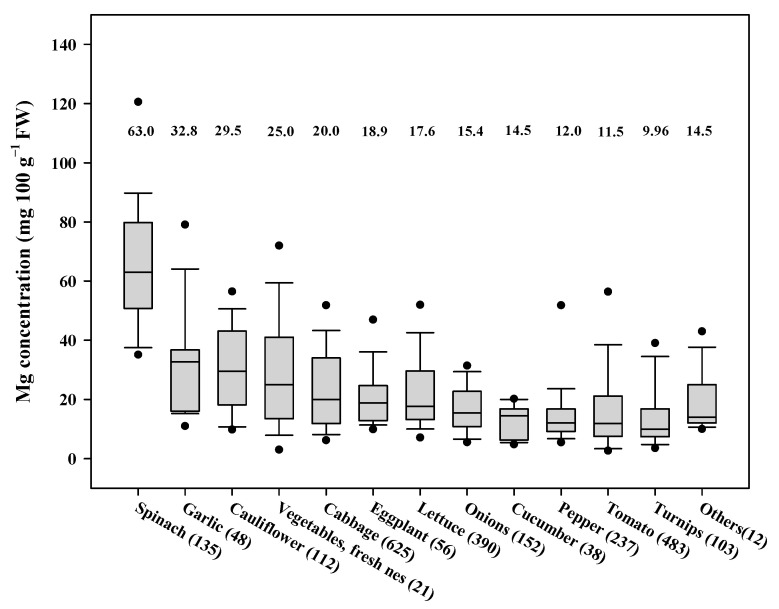
Mg content of major vegetables based on a global meta-analysis. The data represent mean values [expressed on fresh weight basis] with 95% bootstrap confidence intervals, and the sample numbers used for analysis are shown in parenthesis. The solid line passing through the box indicate median. According to FAO datasets, ‘Vegetables, fresh nes’ included in the figure represents: bamboo shoots (*Bambusa* spp.), beets, chards (*Beta vulgaris* L.), capers (*Capparis spinosa* L.), cardoons (*Cynara cardunculus* L.), celery (*Apium graveolens* L.), chervil (*Anthriscus cerefolium* L.), cress (*Lepidium sativum* L.), fennel (*Foeniculum vulgare* L.), horseradish (*Cochlearia armoracia* L.), marjoram, sweet (*Majorana hortensis* L.), oyster plant (*Tragopogon porrifolius* L.), parsley (*Petroselinum crispum* L.), parsnips (*Pastinaca sativa* L.), radish (*Raphanus sativus* L.), rhubarb (*Rheum* spp.), rutabagas, swedes (*Brassica napus* L.), savory (*Satureja hortensis* L.), scorzonera (*Scorzonera hispanica* L.), sorrel (*Rumex acetosa* L.), soybean sprouts tarragon (*Artemisia dracunculus* L.), watercress (*Nasturtium officinale* L.). ‘Others’ included in the figure represents other vegetables that are not identified separately because of their low importance (consumption and market share) internationally.

A global meta-analysis of vegetable Mg content (*n* = 2412, 235 publications from year 1940 to 2018) estimated global weighted average vegetable Mg content to be 19.3 mg 100 g^−1^ FW, and it varied from 9.96 to 63.0 mg 100 g^−1^ FW among different vegetable species analyzed (Figure 1). The Mg content of spinach, garlic, cauliflower and cabbage were higher than of the mean global weighted average.

**Figure 2:**
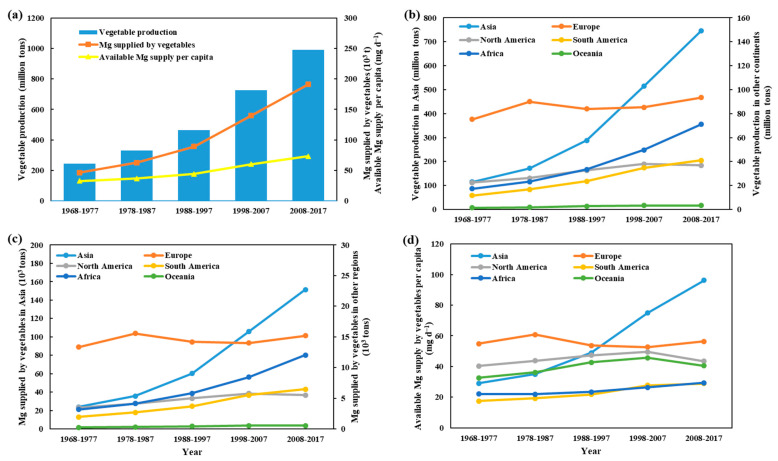
Annual vegetable production- global (**a**) and in different continents (**b**); annual vegetable Mg production- global (**a**) and in 6 continents (**c**); vegetable Mg supply per capita- global (**a**) and in different continents (**d**) in the past 50 years (1968–2017).

On a global scale, the average vegetable production and the Mg supplied by vegetables from 1968–1977 to 2008–2017 period were increased by 308% and 314%, respectively (Figure 2a). During the same period, vegetable-Mg supply per capita was increased by 125% (Figure 2a). Today, vegetable-Mg supply per capita globally has reached 73.0 mg day^−1^. Large variation in vegetable production and total and per capita vegetable-Mg supply exist among the six continents. Vegetable production and vegetable-Mg supply in Asia during 2008–2017 period accounted for 75.2% and 80.0% of the world, respectively. Whilst those values are only 7.17% and 6.40% in Africa (Figure 2b,c). Notably, three continents with the fastest growth rates in vegetable production and vegetable-Mg supply over the past 50 years were in developing regions—Asia, Africa and South America. The vegetable-Mg supply per capita also varied greatly among six continents, ranging from 28.9 to 96.2 mg day^−1^ in 2008–2017 period. Equally concerningly, a significant downward trend in vegetable-Mg supply per capita has observed in North America and Oceania (Figure 2d).

The weighted average vegetable Mg content in 6 continents also vary widely, from 15.3 (North America) to 20.3 (Asia) mg 100 g^−1^ FW (Table 1). This is largely attributed to difference in vegetable species planted in different continents. For example, the proportion of high Mg vegetable (>30 mg 100 g^−1^ FW) production in Asia (6.29%) was considerably higher than that in other continents (0.46–1.64%). A similar trend was evident for vegetable Mg supplied by high Mg vegetables and Mg RDA as well (Table 1).

### 3.2. Current Health and Economic Burden Attributed to Mg Deficiency

Based on the reported health conditions attributed to Mg deficiency, DALYs approach showed that Mg malnutrition of Chinese adult population results in a loss of 1.91 million DALYs per year (Table 2). Among the 3 major diseases, the type 2 diabetes contributed the most YLD loss and the stroke contributed the most YLL due to the relative proportion of population affected by those heath conditions caused by Mg deficiency. The economic burden attributed to Mg deficiency is estimated to be about 15.8 billion dollars (as high as 0.14% of GDP of China in 2017), with stroke, heart failure and type 2 diabetes accounting for 14.9, 6.58, and 78.5%, respectively (Table 2).

### 3.3. Health and Economic Burden Attributed to Mg Deficiency under Different Mg Availability Scenarios

**Figure 3:**
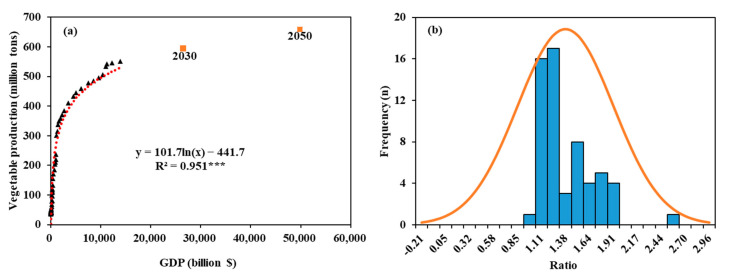
The relationship between gross domestic product (GDP) and vegetable production (**a**) and the improvement ratio of vegetable Mg content by Mg biofortification (**b**). The black triangles represent values from 1960–2018, the orange square represent predicted values in year 2030 and 2050. The predicted GDP of China in 2030 and 2050 are 26,499 and 49,853 billion dollars, respectively [53]. The median value of vegetable Mg content improvement ratio by Mg biofortification was 1.22 based on meta-analysis (Formula (3)). *** indicates significant difference at *p* < 0.001.

Figure 3 provides the basic parameters for scenario analysis. A relationship between GDP and vegetable production was simulated by a logarithmic equation (Figure 3a). According to GDP in the years 2030 and 2050 [53], the predicted vegetable production in 2030 and 2050 are 595 and 659 million tons, respectively, which is 8.93 and 20.7% higher than that reported for 2017. Based on further analysis of vegetable Mg content, we found that vegetable Mg content could be improved by 22% on average through biofortification (Figure 3b).

**Figure 4:**
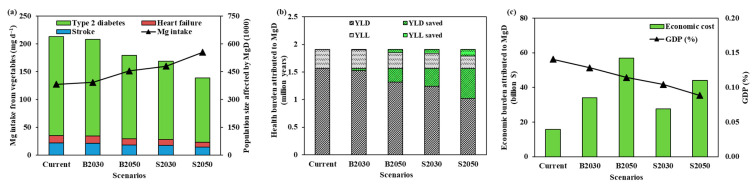
The scenarios analysis of Mg intake from vegetables (Formula (4)), number of cases attributed to Mg deficiency (**a**), DALYs lost, DALYs saved (**b**) (Formula (5)), economic burden attributed to Mg deficiency and its percentage to GDP (**c**). B2030 and B2050 represent business as usual vegetable productions in 2030 and 2050 (based on (**a**)), and S2030 and S2050 represent improving vegetable Mg contents by 22% through biofortification (based on (**b**)).

The dietary Mg intake met by vegetables was increased by 2.80 to 45.1% in 4 possible scenarios. With biofortification the dietary Mg intake met by vegetables reaches 185 mg day^−1^ in S2050. Compared to the current scenario, the number of cases (including deaths and disabilities in 3 major diseases) due to Mg deficiency were significantly decreased (2.45% to 34.8%) in all scenarios (Figure 4a). The DALYs lost (YLD + YLL) were also decreased from 1.91 to 1.24 million years in S2050. In all DALYs saved, YLD and YLL saved account for 82.0 and 18.0%, respectively (Figure 4b). The economic burden arising from the three major disorders caused by Mg deficiency, however, was found to be increased in all future scenarios, though the percentage of GDP loss associated the health conditions showed a decreasing trend (from 0.14% to 0.09%) (Figure S1 and Figure 4c).

## 4. Discussion

Mg is an important biologically active mineral needed by all living cells. It is a critical nutrient for carbohydrate metabolism, protein and nucleic acid synthesis [1,2,3], and cardiovascular and muscle functions [5,7]. Mg plays an important role in glucose metabolism and insulin resistance, and chronic Mg deficiency is frequently associated with type 2 diabetes and muscular dysfunction [54]. Prolonged hypomagnesemia has been implicated in disorders of vascular smooth muscles and endothelial functions, triggering atherosclerosis and subsequently stroke and cardiac failure [55]. Several lines of evidence showed activation of oxidative stress, lipid peroxidation and inflammatory responses under Mg deficiency, which are thought to be mediated by Mg-dependent modulation of intracellular Ca^2+^ concentration, release of neurotransmitters and other factors involved in immune responses [56]. Mg deficiency renders cardiovascular system particularly vulnerable to such immune and inflammatory responses, causing cardiovascular diseases. Long-term large prospective cohort studies and clinical trials have clearly established an inverse relationship between dietary Mg intake and the incidence of type 2 diabetes, stroke, and heart failure [5,6,10]. Also, independent studies have shown reduction in both systolic and diastolic blood pressure following Mg supplementation [57]. Since plant food forms one of the main sources of dietary Mg, here we discuss the status and reasons for the large inter-continental variation in vegetable Mg supply observed in this study, and its health and economic implications on hypomagnesemia-attributed cases of type 2 diabetes, stroke, and heart failure. Also, the potential of Mg biofortification of vegetables as a strategy to reduce the risk of these three diseases and associated economic and health impact in China has been explored.

Many vegetables are rich in Mg and other minerals needed for human health. Vegetable production and vegetable Mg supply made a gradual but impressive increase globally over the past fifty years (Figure 2a). An important observation here is that the bulk of the gain in vegetable production has occurred in Asia, with a much weaker upward trend noticed for Africa and South America (Figure 2b). Vegetable production, however, remained static in Europe and North America for the same period. This is not surprising as Asia accounts for most of the population growth and also witnessed a remarkable lift in economic development in the past five decades, which spurred the demand for nutritious and quality food. This also resulted in a substantial improvement in per capita vegetable Mg supply in Asia, especially in the last three decades (Figure 2d). This is largely due to the increased vegetable production and Mg-rich vegetables cultivated (Table 1). The situation, however, remains bleak in other continents with little progress made in per capita vegetable Mg supply in the past five decades, and this forms a major hidden hunger globally (Figure 2d). The findings of this study thus underscore the importance of increasing vegetable Mg content to alleviate hypomagnesemia and the attended health and economic loss.

Globally economic development of a country is accompanied by increased intake of meat, dairy and processed food, which are all inherently low in Mg [58]. Also, economic development is strongly correlated with many lifestyle disorders such as obesity, diabetes, cardiovascular diseases, and inflammatory and immune disorders. Despite the abundance of nutrient-rich food in developed countries, increased incidence of several nutritional disorders is widespread in Europe and North America [59]. For instance, up to 15% of American population has some degree Mg deficiency, and dietary surveys and epidemiological studies have shown that dietary intake of Mg was declining from ~500 mg/day to about 225 mg/day over the past hundred years in the United States [13]. Such trends are also evident in other western countries, and it is attributed to excessive consumption of highly processed grains, imbalanced diet, increased use of chemical fertilizers, reduced crop diversity and growing alcohol consumption [11,12,59]. Surprisingly, though vegetable Mg production remains relatively low in Africa (Figure 2), Mg deficiency in African population is unlikely to be a major problem, mainly because unprocessed whole grain cereals and millets are the major source of food for energy and this assures adequate daily intake of Mg [58]. Further, at least for now, increasing vegetable production is not a priority for most African and South American countries, as they are still in the process of increasing cereal production to ensure food security. However, it is important to recognize that, along with Mg, adequate provision of other micro-nutrients is required, and thus nutrient-rich vegetables and fruit production has to be included in the food security matrix in Africa.

From the above discussion, it is apparent that Mg deficiency causes or exacerbates several serious health conditions across all continents and among them hypomagnesemia-induced type 2 diabetes, stroke and heart failure are major health issues of China. We have used DALYs to quantify various elements of health loss due to cases of these diseases attributed to Mg deficiency in China. Among the three diseases, type 2 diabetes accounted for the largest number of disabilities and YLD loss, which was about 390 times more than that of stroke (Table 2). Stroke, however caused the highest number of death and YLL. This reflects the large incidence of chronic hypomagnesemia in Chinese population. Despite stroke accounting for the greatest mortality and YLL, it is hypomagnesemia-related type 2 diabetes that causes the highest economic burden, estimated to be $12.4 billion. The health and economic impact of these three health conditions attributed to hypomagnesemia can be readily alleviated by increased dietary intake of Mg [60,61,62]. Previous studies have shown decreased incidence and better management of these three diseases with Mg supplementation [10].

Being the rich source of dietary Mg, increased consumption of Mg-rich vegetables offers a viable strategy to alleviate this health burden. Vegetables are a particularly attractive candidate for this purpose because they contain significant quantities of Mg and also provide numerous other nutrients essential for human health simultaneously. However, unlike appearance qualities such as color and shape, flavour, processing qualities, and disease and pest resistance, improving nutritional quality is not a priority for vegetable breeding due to yield and quality traits trade-offs [63]. Therefore, vegetable Mg content can be increased further by biofortification through different crop management practices and/or breeding, allowing the production of a variety of Mg-enriched vegetables, which offer choices for customers with different preferences and production options. Biofortification has been successfully achieved for Zn, Fe, Ca, I, Si and Se nutrients in wheat, rice and brassica [25,26,27,28]. Assuming that the current vegetable crop composition scenario will remain as it is but will continue the trajectory of increased production in China, a scenario analysis showed an appreciable reduction in type 2 diabetes, stroke and heart failure by 2050. However, the rate of decline of these diseases can be greatly accelerated with biofortified vegetables with increased Mg content (Figure 4a). The positive health and economic outcome of widespread use of Mg biofortified vegetables goes much beyond the three diseases considered here. For instance, Mg malnutrition of Chinese adult population incurs a loss of 1.91 million DALYs, with an economic burden estimated to be 15.8 billion dollars (~0.14% of GDP). Considering the enormity of health and economic cost involved, we propose the need for a nation-wide strategy to increase vegetable production and vegetable Mg biofortification to improve per capita vegetable Mg supply. Since consumer choice of vegetables is largely determined by dietary preferences and cultural backgrounds, successful delivery of increased per capita vegetable Mg supply will require: (1) careful choice of vegetables suitable for different regions, (2) genetic and crop production innovations to maximize vegetable production, including remodeling vegetable cultivation to increase the proportion of vegetables with high Mg content; (3) biofortification to boost vegetable Mg content; (4) minimise vegetables loss during harvesting, storage, transportation and processing. Biofortification through innovative agronomic practices is expected to make a significant positive impact on vegetable Mg supply, but there are limitations to this technology. Not all vegetables are amenable to biofortification through agronomy innovations. Thus, considerable research to identify vegetables and the crop production methodologies suited for biofortification is critical. We believe that implementing the strategy outlined above will be a cost-effective and technologically feasible option for decreasing hypomagnesemia and the associated health and economic burden in China and elsewhere relatively rapidly.

## 5. Conclusions

Hypomagnesemia is a significant human nutritional disorder that occurs widely in both developed and developing countries globally. Several health conditions such as cardiovascular diseases, neurological disorders, type 2 diabetes, immunological and inflammatory diseases are associated with hypomagnesemia. Vegetables are the principal source of dietary Mg, but vegetable availability and per capita vegetable-Mg supply remain grossly inadequate to address this disorder. Also, per capita vegetable Mg supply vary greatly between continents with Asia recording the highest and Oceania and South America the lowest amount. A case study of hypomagnesemia-attributed stroke, type 2 diabetes, and heart failure in China showed a combined loss of $15.8 billion annually with type 2 diabetes accounting for much of the economic burden. A scenario analysis revealed greater vegetable Mg supply through judicious choice of Mg-rich vegetables and their increased production as well as Mg biofortification to boost vegetable Mg content greatly reduce the health and economic burden of these diseases. This innovative approach of increased dietary intake of Mg is cost-effective, technologically less demanding, and can be tailored to meet regional requirements.

## Figures and Tables

**Table 1:** Weighted average vegetable Mg content, high Mg vegetable production and percentage of recommended daily allowance of Mg supplied by vegetables in 6 continents.

	Asia	Europe	North America	South America	Oceania	Africa
Vegetable Mg content (mg 100 g^−1^ FW)	20.3	16.1	15.3	15.8	17.1	16.9
High Mg vegetables production (%) ^a^	6.29	1.62	1.64	1.46	0.46	1.00
Vegetable Mg supplied by high Mg vegetables (%)	14.9	4.54	5.41	3.46	1.58	2.19
RDA (%)	25.7	14.3	10.1	7.68	9.54	7.37

^a^ Vegetables were classified into 3 groups based on their Mg content. High Mg vegetables: >30 mg 100 g^−1^ FW (include spinach and garlic); Medium Mg vegetables: Mg content from 20 to 30 mg 100 g^−1^ FW (include cauliflower, vegetable fresh nes and cabbage); Low Mg vegetables: Mg content < 20 mg 100 g^−1^ FW (include eggplant, lettuce, onions, cumumber, pepper, tomato, turnips and others).

**Table 2:** Health and economic burden of Mg malnutrition, a case study in China.

Major Disease Types	Number of Disabilities Due to MgD ^a^	Years Lived with Disability (YLD) ^b^	Number of Deaths Due to MgD ^c^	Years of Life Lost (YLL) ^d^	Annual Healthcare Service Costs ($) ^e^	Direct Economic Burden (Billion $)	Indirect Economic Burden (Billion $) ^f^	Economic Burden (Billion $)	GDP (%) ^g^
Stroke	31,903	3799	33,983	293,214	1001	0.07	2.30	2.36	0.02
Heart failure	32,614	77,935	7412	33,902	4289	0.17	0.86	1.04	0.01
Type 2 diabetes	532,059	1,483,387	2011	15,860	1502	0.80	11.6	12.4	0.11
Sum	596,576	1,565,121	43,406	342,976	- -	1.04	14.8	15.8	0.14

^a^ MgD means Mg deficiency. Calculated based on data of target population size from China Health and Nutrition Survey (CHNS) and National Bureau of Statistics of China [40], the incidence rate of 3 major diseases [41,42,43] and their attribution to Mg deficiency [10]. ^b^ Calculated based on number of disabilities due to Mg deficiency, the disability weight of the 3 major diseases [44] and their duration [40,45] (Formula (1)). ^c^ Calculated based on data of target population size from China Health and Nutrition Survey (CHNS) and National Bureau of Statistics of China [40], the mortality rate of 3 major diseases [40,41,42,46,47] and their attribution to Mg deficiency [10]. ^d^ Calculated based on the number of deaths due to Mg deficiency and the remaining life expectancy of 3 major diseases [40,48,49] (Formula (2)). ^e^ The annual healthcare service costs of 3 major diseases per patient [50,51,52]. ^f^ Indirect economic burden was calculated by DALYs lost multiplied by GDP per capita. The GDP per capita in China in 2017 was 7733 $ based on the World Bank. ^g^ GDP in China in 2017 was 12,310 billion $ based on the World Bank.

## Supplementary Materials

The following are available online at www.mdpi.com/article/10.3390/foods10112513/s1, Table S1: Production quantities of top 12 vegetables which account for 88% of total vegetable productions and their moisture contents (Based on FAO and USDA); Figure S1: Healthcare spending of GDP in 2030 and 2050 in China predicted by its historical trends (2000–2017) and predicted annual healthcare cost of stroke, heart failure and type 2 diabetes in 2030 and 2050; Extended reference list: 235 published references for Mg contents in different vegetable species.
