# Supplementary material for: Increased Provision of Bioavailable Mg through Vegetables Could Significantly Reduce the Growing Health and Economic Burden Caused by Mg Malnutrition
Source: Foods. 2021 Oct 20;10(11):2513. doi: 10.3390/foods10112513 (PMC8620491; doi:10.3390/foods10112513)
Supplement: Supplementary file 1 [file foods-10-02513-s001.zip › foods-1404510-supplementary.pdf]

**Supplementary Materials for "Increased Provision of Bioavailable Mg Through Vegetables Could Significantly Reduce the Growing Health and Economic"**

**Table S1.** Production quantities of top 12 vegetables which account for 88% of total vegetable productions and their moisture contents (Based on FAO and USDA).

| | Production (million tons) | Production (%) | Moisture content (%) |
|---|---|---|---|
| Vegetables, fresh nes | 298 | 27.33 | -- |
| Tomatoes | 182 | 16.74 | 94.5 |
| Onions | 96.8 | 8.89 | 89.1 |
| Cucumbers | 75.2 | 6.91 | 95.2 |
| Cabbages | 69.4 | 6.37 | 92.6 |
| Eggplants | 54.1 | 4.97 | 92.0 |
| Carrots | 40.0 | 3.67 | 95.3 |
| Peppers | 36.8 | 3.38 | 93.9 |
| Garlic | 28.5 | 2.62 | 59.0 |
| Lettuce | 27.3 | 2.50 | 95.6 |
| Cauliflowers | 26.5 | 2.43 | 90.7 |
| Spinach | 26.3 | 2.41 | 91.4 |

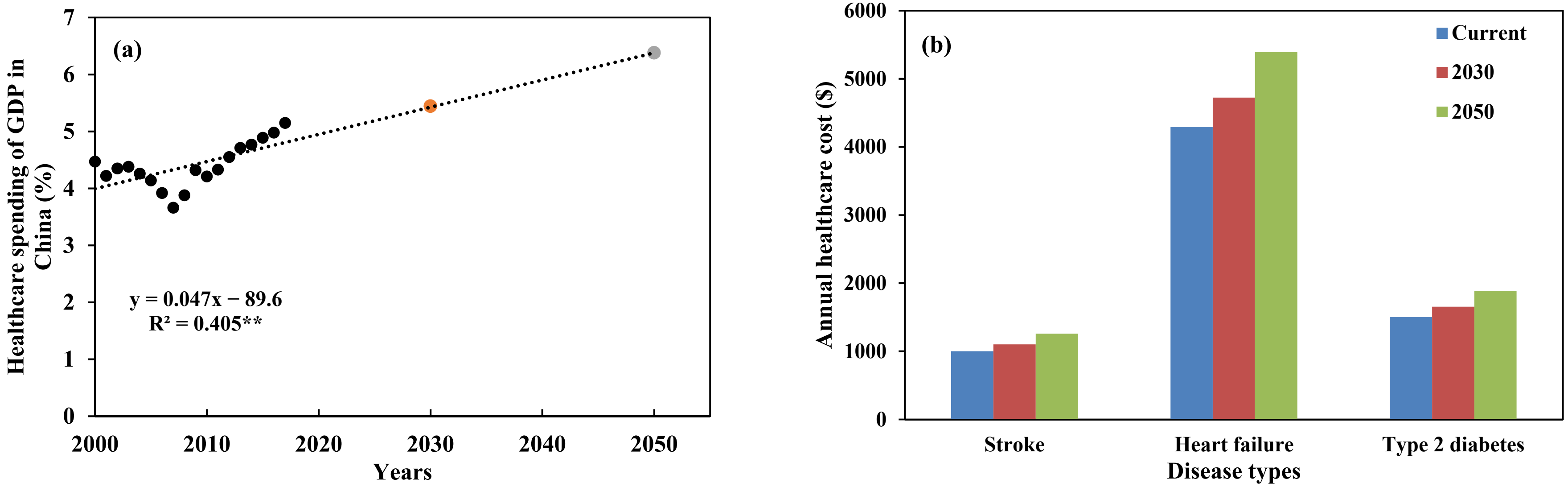


**Figure S1.** Healthcare spending of GDP in 2030 and 2050 in China (a) predicted by its historical trends (2000-2017) and predicted annual healthcare cost (b) of stroke, heart failure and type 2 diabetes in 2030 and 2050. ** indicates significant difference at $P < 0.01$.

**Extended reference list:** 235 published references for Mg concentrations in different vegetable species.


[1]. Acikgoz, F.E.; Deveci, M. Comparative analysis of vitamin C, crude protein, elemental nitrogen and mineral content of canola greens (*Brassica napus* L.) and kale (*Brassica oleracea* var. acephala). *African J. Biotechnol.* **2011**, *10*, 19385–19391.

[2]. Adams, P.; Graves, C.J.; Winsor, G.W. Some responses of cucumber, grown in beds of peat, to N, K and Mg. *J. Hortic. Sci.* **1992**, *67*, 877–884.

[3]. Adekiya, A.O.; Agbede, T.M.; Aboyeji, C.M.; Dunsin, O.; Simeon, V.T. Effects of biochar and poultry manure on soil characteristics and the yield of radish. *Sci. Hortic.* **2019**, *243*, 457–463.

[4]. Adeyeyei, E.I.; Otokiti, M.K.O. Proximate composition and some nutritionally valuable minerals of two varieties of *capsicum annum* L. (bell and cherry peppers). *Discov. Innov.* **1999**, *11*, 75–81.

[5]. Akinwande, B.A.; Olatunde, S.J. Comparative evaluation of the mineral profile and other selected components of onion and garlic. *Int. Food Res. J.* **2015**, *22*, 332–336.

[6]. Alabi, D.A. Effects of fertilizer phosphorus and poultry droppings treatments on growth and nutrient components of pepper (*Capsicum annuum* L). *African J. Biotechl.* **2006**, *5*, 671–677.

[7]. Al-Bataina, B.A.; Maslat, A.O.; Al-Kofahi, M.M. Element analysis and biological studies on ten oriental spices using XRF and AMES test. *J. Trace Elem. Med. Biol.* **2003**, *17*, 85–90.

[8]. Al-Jasass, F.M.; Al-Jasser, M.S. Chemical composition and fatty acid content of some spices and herbs under Saudi Arabia conditions. *Sci. World J.* **2012**, *2012*, 18–23.

[9]. Antunes, L.F. de S.; Scoriza, R.N.; Silva, D.G. da; Correia, M.E.F. Produção e eficiência de composto orgânico gerado pela atividade de gongolos. *Cienc. Rural* **2016**, *46*, 815–819.

[10]. Anunciação, D.S.; Leao, D.J.; de Jesus, R.M.; Ferreira, S.L.C. Use of multivariate analysis techniques for evaluation of analytical data-determination of the mineral composition of cabbage (*Brassica oleracea*). *Food Anal. Methods* **2011**, *4*, 286–292.

[11]. Araújo, E.M.; de Lima, M.D.; Barbosa, R.; Alleoni, L.R.F. Using machine learning and multi-element analysis to evaluate the authenticity of organic and conventional vegetables. *Food Anal. Methods* **2019**, *12*, 2542–2554.

[12]. Aremu, C.Y.; Udoessien, E.I. Chemical estimation of some inorganic elements in selected tropical fruits and vegetables. *Food Chem.* **1990**, *37*, 229–234.

[13]. Ariyama, K.; Horita, H.; Yasui, A. Application of inorganic element ratios to chemometrics for determination of the geographic origin of Welsh onions. *J. Agric. Food Chem.* **2004**, *52*, 5803–5809.

[14]. Ashfaq, F.; Butt, M.S.; Nazir, A.; Jamil, A. Compositional analysis of Pakistani green and red cabbage. *Pakistan J. Agric. Sci.* **2018**, *55*, 191–196.

[15]. Assimakopoulou, A.; Kotsiras, A.; Nifakos, K. Incidence of lettuce tipburn as related to hydroponic system and cultivar. *J. Plant Nutr.* **2013**, *36*, 1383–1400.

[16]. Awad, Y.M.; Lee, S.E.; Ahmed, M.B.M.; Vu, N.T.; Farooq, M.; Kim, I.S.; Kim, H.S.; Vithanage, M.; Usman, A.R.A.; Al-Wabel, M.; et al. Biochar, a potential hydroponic growth substrate, enhances the nutritional status and growth of leafy vegetables. *J. Clean. Prod.* **2017**, *156*, 581–588.

[17]. B. Yan; J. Han; Z. Chen; M. Cai; Y. Wei; H. Wang; H. Wang. Effects of magnesium and potassium fertilization on distribution and ratios of Ca，Mg and K in soil liquid and solid phase in greenhouse. *Agri Res Arid Areas*. **2017**, *35*, 181-186. (in Chinese with English abstract)

[18]. Bakht, J.; Khan, L.; Shafi, M. Phytoaccumulation of heavy metals and protein expression by different vegetables collected from various parts of Khyber Pukhtunkhawa Province, Pakistan. *Sains Malaysiana* **2016**, *45*, 167–176.

[19]. Barickman, T.C.; Horgan, T.E.; Wheeler, J.R.; Sams, C.E. Elevated levels of potassium in greenhouse-grown red romaine lettuce impacts mineral nutrient and soluble sugar concentrations. *HortScience* **2016**, *51*, 504–509.

[20]. Barta, D.J.; Tibbitts, T.W. Calcium localization in lettuce leaves with and without tipburn: comparison of controlled-environment and field-grown plants. *J. Am. Soc. Hortic. Sci.* **1991**, *116*, 870–875.

[21]. Bazewicz-Woźniak, M.; Kęsik, T.; Wach, D.; Konopiński, M. The influence of conservation tillage on the mineral elements content in soil and chemical composition of onion. *Acta Sci. Pol. Hortorum Cultus*

**2008**, *7*, 61–72.
[22]. Berard, L.S. Effects of nitrogen fertilization on stored cabbage. III. Changes with time and distribution in outer-head leaves of the mineral contents. *J. Hortic. Sci.* **1990**, *65*, 417–422.
[23]. Bernardo, A.; Martínez, S.; Álvarez, M.; Fernández, A.; López, M. The composition of two Spanish pepper varieties (fresno de la vega and benavente-los valles) in different ripening stages. *J. Food Qual.* **2008**, *31*, 701–716.
[24]. Bettoni, M.M.; Mogor, Á.F.; Pauletti, V.; Goicoechea, N.; Aranjuelo, I.; Garmendia, I. Nutritional quality and yield of onion as affected by different application methods and doses of humic substances. *J. Food Compos. Anal.* **2016**, *51*, 37–44.
[25]. Bhardwaj, H.L.; Hamama, A.A.; Rangappa, M. Characterization of nutritional quality of canola greens. *HortScience.* **2003**, *38*, 1156–1158.
[26]. Bhattacharjee, S.; Dasgupta, P.; Paul, A.R.; Ghosal, S.; Padhi, K.K.; Pandey, L.P. Mineral element composition of spinach. *J. Sci. Food Agric.* **1998**, *77*, 456–458.
[27]. Bibak, A.; Stürup, S.; Knudsen, L.; Gundersen, V. Concentrations of 63 elements in cabbage and sprouts in Denmark. *Commun. Soil Sci. Plant Anal.* **1999**, *30*, 2409–2418.
[28]. Bierman, P.M.; Rosen, C.J. Sewage sludge incinerator ash effects on soil chemical properties and growth of lettuce and corn. *Commun. Soil Sci. Plant Anal.* **1994**, *25*, 2409–2437.
[29]. Blasco, B.; Ríos, J.J.; Sánchez-Rodríguez, E.; Rubio-Wilhelmi, M.M.; Leyva, R.; Romero, L.; Ruiz, J.M. Study of the interactions between iodine and mineral nutrients in lettuce plants. *J. Plant Nutr.* **2012**, *35*, 1958–1969.
[30]. Blatt, C.R.; McRae, K.B. Comparison of four organic amendments with a chemical fertilizer applied to three vegetables in rotation. *Can. J. Plant Sci.* **1998**, *78*, 641–646.
[31]. Bogoescu, M.I. Influence of correcting the acidity of soil and usage of potassium fertilizers on physiological, biochemical characteristics and storage capacity of the autumn cabbage. *Not. Bot. Horti Agrobot. Cluj-Napoca* **2017**, *45*, 491–497.
[32]. Bong, Y.S.; Shin, W.J.; Gautam, M.K.; Jeong, Y.J.; Lee, A.R.; Jang, C.S.; Lim, Y.P.; Chung, G.S.; Lee, K.S. Determining the geographical origin of Chinese cabbages using multielement composition and strontium isotope ratio analyses. *Food Chem.* **2012**, *135*, 2666–2674.
[33]. Bouzari, A.; Holstege, D.; Barrett, D.M. Mineral, fiber, and total phenolic retention in eight fruits and vegetables: A comparison of refrigerated and frozen storage. *J. Agric. Food Chem.* **2015**, *63*.
[34]. Buczkowska, H.; Michałojć, Z. Comparison of qualitative traits, biological value, chemical compounds of sweet pepper fruit. *J. Elem.* **2012**, *17*, 367–377.
[35]. Bystrická, J.; Kovarovič, J.; Lenková, M.; Horváthová, J.; Končeková, L.; Halmová, D.; Lidiková, A. The content of polyphenols, antioxidant activity and macroelements in selected garlic varieties. *J. Microbiol. Biotechnol. Food Sci.* **2018**, *8*, 738–740.
[36]. C. Huang; Z. Wang; J. Li; H. Wang; J. Wang; F. Chong; M. Pu. Magnesium deficiency in Chinese cabbage and analysis of related nutritional elements. *Bull. Agric. Sci. Technol*. **2019**, 188-191. (in Chinese with English abstract)
[37]. C. Xiao; Z. Yang. Effects of different calcium and magnesium fertilizers on Yield and quality of vegetables . *J. Changjiang. Veg*. **2000**, 27-29. (in Chinese with English abstract)
[38]. Cardarelli, M.; Rouphael, Y.; Muntean, D.; Colla, G. Growth, quality index, and mineral composition of five ornamental cabbage cultivars grown under different nitrogen fertilization rates. *HortScience.* **2015**, *50*, 688–693.
[39]. Cardoso, M.O.; Oliveira, A.P. de; Pereira, W.E.; Souza, A.P. de Growth, nutrition and yield of eggplant as affected by doses of cattle manure and magnesium thermophosphate plus cow urine. *Hortic. Bras.* **2009**, *27*, 307–313.
[40]. Chevaux, K.A.; Jackson, L.; Elena Villar, M.; Mundt, J.A.; Commisso, J.F.; Adamson, G.E.; McCullough, M.M.; Schmitz, H.H.; Hollenberg, N.K. Proximate, mineral and procyanidin content of certain foods and beverages consumed by the Kuna Amerinds of Panama. *J. Food Compos. Anal.* **2001**, *14*, 553–563.
[41]. Çikili, Y.; Samet, H.; Dursun, S. Cadmium toxicity and its effects on growth and metal nutrient ion accumulation in Solanaceae plants. *Tarim Bilim. Derg.* **2016**, *22*, 576–587.
[42]. Citak, S.; Sonmez, S. Influence of organic and conventional growing conditions on the nutrient

contents of white head cabbage (*Brassica oleracea* var. capitata) during two successive seasons. *J. Agric. Food Chem.* **2010**, *58*, 1788–1793.
[43]. Citak, S.; Sonmez, S. Mineral contents of organically and conventionally grown spinach (Spinacea oleracea L.) during two successive seasons. *J. Agric. Food Chem.* **2009**, *57*, 7892–7898.
[44]. Cruz, R.; Morais, S.; Mendes, E.; Pereira, J.A.; Baptista, P.; Casal, S. Improvement of vegetables elemental quality by espresso coffee residues. *Food Chem.* **2014**, *148*, 294–299.
[45]. Cutcliffe, J.A. Effects of added limestone and potassium on yield and storage losses of cabbage. *Can. J. Plant Sci.* **1984**.
[46]. Cvetković, B.R.; Pezo, L.L.; Mišan, A.; Mastilović, J.; Kevrešan, Ž.; Ilić, N.; Filipčev, B. The effects of osmotic dehydration of white cabbage on polyphenols and mineral content. *Lwt* **2019**, *110*, 332–337.
[47]. Czekała, J.; Jezierska, A.; Krzywosadzki, A. Effect of nitrogen fertilization on P, K, Mg, Ca and S content in soil and edible parts of white cabbage. *J. Elem.* **2011**, *16*, 169–176.
[48]. D. Han; D. Wang; P. Huang; J. Duan; R. Ge; W. Zhou. Effects of different morphology magnesium on yield and quality of 'Zaoshu 5'Chinese cabbage. *Acta. Hortic. Sin.* **2010**, *37*, 1655-1660. (in Chinese with English abstract)
[49]. de Oliveira, R.A.; Brunetto, G.; Loss, A.; Gatiboni, L.C.; Kürtz, C.; Müller Júnior, V.; Lovato, P.E.; Oliveira, B.S.; Souza, M.; Comin, J.J. Cover crops effects on soil chemical properties and onion yield. *Rev. Bras. Cienc. do Solo* **2016**, *40*, 1–17.
[50]. dos Santos, A.M.P.; Lima, J.S.; Anunciação, D.S.; Souza, A.S.; dos Santos, D.C.M.B.; Matos, G.D. Determination and evaluation employing multivariate analysis of the mineral composition of broccoli (*Brassica oleracea* L. var. Italica). *Food Anal. Methods* **2013**, *6*, 745–752.
[51]. Dungan, R.S.; Dees, N.H. Use of spinach, radish, and perennial ryegrass to assess the availability of metals in waste foundry sands. *Water. Air. Soil Pollut.* **2007**, *183*, 213–223.
[52]. Dursun, A.; Turan, M.; Ekinci, M.; Gunes, A.; Ataoglu, N.; Aslihan, E.; Yildirim, E. Effects of boron fertilizer on tomato, pepper, and cucumber yields and chemical composition. *Commun. Soil Sci. Plant Anal.* **2010**, *41*, 1576–1593.
[53]. Dzida, K.; Jarosz, Z.; Michałojć, Z.; Nurzyńska-Wierdak, R. The influence of diversified nitrogen and liming fertilization on the chemical composition of lettuce. *Acta Sci. Pol. Hortorum Cultus.* **2012**.
[54]. Eftekhari Zadeh, E.; Feghhi, S.A.H.; Bayat, E. Determination of the major, minor, and trace element mass fractions in Iranian cement by INAA and WDXRF. *Radiochem*. **2016**.
[55]. Erfani, F.; Hassandokht, M.R.; Jabbari, A.; Barzegar, M. Effect of cultivar on chemical composition of some Iranian spinach. *Pakistan J. Biol. Sci.* **2007**.
[56]. F. Liu; Z. Han. Contents variation and influence appraisal of U, Th, K, Fe, Mo, Mg and Zn of fly ash soil and vegetables. *Coal. Mine. Environ. Prot*. **2002**, 50-53. (in Chinese with English abstract)
[57]. F. Xu. Effects of organic fertilizer input on Ca, Mg, Mo, Li content and distribution in spinach. *North. Hortic.* **2012**, 144-145. (in Chinese with English abstract)
[58]. Fältmarsch, R.; Österholm, P.; Jacks, G. Chemical composition of cabbage (*brassica oleracea* L. var. capitata) grown on acid sulfate soils. *J. Plant Nutr. Soil Sci.* **2010**, *173*, 423–433.
[59]. Ferrarezi, R.S.; Testezlaf, R. Performance of wick irrigation system using self-compensating troughs with substrates for lettuce production. *J. Plant Nutr.* **2016**, *39*, 147–161.
[60]. Flores, R.A.; Borges, B.M.M.N.; Almeida, H.J.; Prado, R.D.M. Growth and nutritional disorders of eggplant cultivated in nutrients solutions with suppressed macronutrients. *J. Plant Nutr.* **2015**, *38*.
[61]. Francisco, M.; Velasco, P.; Lema, M.; Cartea, M.E. Genotypic and environmental effects on agronomic and nutritional value of Brassica rapa. *Agron. J.* **2011**, *103*, 735–742.
[62]. Fujiyama, H. Studies on responses of plants grown on sand dune soil to a nutrient solution applied by drip irrigation. III. Effect of activity ratio of potassium to calcium and magnesium in nutrient solution on lettuce growth. *Soil Sci. Plant Nutr.* **1989**.
[63]. Fung, A.C. Elements in fresh and in frozen spinach and collards. *Anal. Methods. Stat. Anal.* **1978**, *43*.
[64]. Furr, A.K.; Kelly, W.C.; Bache, C.A.; Gutenmann, W.H.; Lisk, D.J. Multielement uptake by vegetables and millet grown in pots on fly ash amended soil. *J. Agric. Food Chem.* **1976**, *24*, 885–888.
[65]. Furr, A.K.; Parkinson, T.F.; Bache, C.A.; Gutenmann, W.H.; Pakkala, I.S.; Lisk, D.J. Elemental content of vegetables and millet grown in potted soil amended with lignite fly ash. *Arch. Environ. Contam. Toxicol.* **1981**, *10*, 647–651.

[66]. G. Song; L. Yang; G. Liu; W. Quan; G. Lu. The effect of calcium on the absorption and distribution of calcium, magnesium and sulfur in cabbage. *J. Shandong. Agric. Univ*. **1998**, 3-5. (in Chinese with English abstract)
[67]. G. Xia; J. Yang. Effects of calcium and magnesium fertilizer on the absorption of mineral elements in tomato plant and fruit. *North. Hortic*. **2005**, 44-45. (in Chinese with English abstract)
[68]. Gamela, R.R.; Costa, V.C.; Pereira-Filho, E.R. Multivariate optimization of ultrasound-assisted extraction procedure for the determination of Ca, Fe, K, Mg, Mn, P, and Zn in pepper samples by ICP-OES. *Food Anal. Methods* **2020**, *13*, 69–77.
[69]. García-Gaytán, V.; Trejo-Téllez, L.I.; Gómez-Merino, F.C.; García-Morales, S.; Tejeda-Sartorius, O.; Ramírez-Martínez, M.; Delgadillo-Martínez, J. Gamma radiation and osmotic potential of the nutrient solution differentially affect macronutrient concentrations, pH and EC in chilhuacle pepper fruits. *J. Radioanal. Nucl. Chem.* **2018**, *315*, 145–156.
[70]. Golubkina, N.A.; Kosheleva, O. V.; Krivenkov, L. V.; Dobrutskaya, H.G.; Nadezhkin, S.; Caruso, G. Intersexual differences in plant growth, yield, mineral composition and antioxidants of spinach (*Spinacia oleracea* L.) as affected by selenium form. *Sci. Hortic.* **2017**, *225*, 350–358.
[71]. Gómez, I.; Navarro Pedreño, J.; Moral, R.; Iborra, M.R.; Palacios, G.; Mataix, J. Salinity and nitrogen fertilization affecting the macronutrient content and yield of sweet pepper plants. *J. Plant Nutr.* **1996**, *19*, 353–359.
[72]. Guerrero, C.C.; Carrasco de Brito, J.; Lapa, N.; Oliveira, J.F.S. Re-use of industrial orange wastes as organic fertilizers. *Bioresour. Technol.* **1995**, *53*, 43–51.
[73]. Güler, S.; Ibrikci, H.; Büyük, G. Effects of different nitrogen rates on yield and leaf nutrient contents of drip-fertigated and greenhouse-grown cucumber. *Asian J. Plant Sci.* **2006**.
[74]. Gülser, F. Effects of ammonium sulphate and urea on $NO_3^-$ and $NO_2^-$ accumulation, nutrient contents and yield criteria in spinach. *Sci. Hortic.* **2005**, *106*, 330–340.
[75]. Gundersen, V.; Bechmann, I.E.; Behrens, A.; Stürup, S. Comparative investigation of concentrations of major and trace elements in organic and conventional Danish agricultural crops. 1. Onions (Allium cepa Hysam) and peas (Pisum sativum Ping Pong). *J. Agric. Food Chem.* **2000**, *48*, 6094–6102.
[76]. Gunes, A.; Inal, A.; Sahin, O.; Taskin, M.B.; Atakol, O.; Yilmaz, N. Variations in mineral element concentrations of poultry manure biochar obtained at different pyrolysis temperatures, and their effects on crop growth and mineral nutrition. *Soil Use Manag.* **2015**, *31*, 429–437.
[77]. Gupta, K.K.; Bhattacharjee, S.; Kar, S.; Chakrabarty, S.; Thakur, P.; Bhattacharyya, G.; Srivastava, S.C. Mineral compositions of eight common spices. *Commun. Soil Sci. Plant Anal.* **2003**, *34*, 681–693.
[78]. Gusman, G.S.; Oliveira, J.A.; Farnese, F.S.; Cambraia, J. Mineral nutrition and enzymatic adaptation induced by arsenate and arsenite exposure in lettuce plants. *Plant Physiol. Biochem.* **2013**, *71*, 307–314.
[79]. Guvenc, I.; Yildirim, E. Increasing productivity with intercropping systems in cabbage production. *J. Sustain. Agric.* **2006**, *28*, 29–44.
[80]. Haciseferoğullari, H.; Özcan, M.; Demir, F.; Çalişir, S. Some nutritional and technological properties of garlic (*Allium sativum* L.). *J. Food Eng.* **2005**, *68*, 463–469.
[81]. Haloi, A.; Thabah, C.R.; Limbu, D.K.; Dkhar, P.S.; Chakraborty, R. Assessment of certain essential elements in some common edibles from dadara and agyathuri villages of kamrup district of assam. *J. Hum. Ecol.* **2010**, *31*, 79–85.
[82]. Hansen, H. Comparison of chemical composition and taste of biodynamically and conventionally grown vegetables. *Qual. Plant. Plant Foods Hum. Nutr.* **1980**, *30*, 203–211.
[83]. Hara, T.; Sonoda, Y. The role of macronutrients in cabbage- head formation: contribution to cabbage-head formation of calcium, magnesium or sulfur supplied at different growth stages. *Soil Sci. Plant Nutr.* **1981**, *27*, 45–54.
[84]. Hopkins, H.; Eisen, J. Mineral elements in fresh vegetables from different geographic areas. *J. Agric. Food Chem.* **1959**, *7*, 633–638.
[85]. Huett, D.O.; Menary, R.C. Effect of aluminium on growth and nutrient uptake of cabbage, lettuce and kikuyu grass in nutrient solution. *Aust. J. Agric. Res.* **1980**, *31*, 749–761.
[86]. Huett, D.O.; Rose, G. Diagnostic nitrogen concentrations for tomatoes grown in sand culture. *Aust. J. Exp. Agric.* **1988**, *28*, 401–409.
[87]. Hussein, A.H.A. Impact of sewage sludge as organic manure on some soil properties, growth, yield

and nutrient contents of cucumber crop. *J. Appl. Sci.* **2009**, *9*, 1401–1411.

[88]. Hwang, S.G.; Chao, H.C.; Lin, H.L. Differential responses of pak choi and edible amaranth to an elevated temperature. *HortScience* **2018**, *53*, 195–199.

[89]. Inthichack, P.; Nishimura, Y.; Fukumoto, Y. Diurnal temperature alternations on plant growth and mineral absorption in eggplant, sweet pepper, and tomato. *Hortic. Environ. Biotechnol.* **2013**, *54*, 37–43.

[90]. Inthichack, P.; Nishimura, Y.; Fukumoto, Y. Effect of potassium sources and rates on plant growth, mineral absorption, and the incidence of tip burn in cabbage, celery, and lettuce. *Hortic. Environ. Biotechnol.* **2012**, *53*, 135–142.

[91]. J. Liu. Bio-effects of magnesium, zinc and boron combination on lettuce nutrition. *J. Chongqing Normal. Univ*. **2003**, 50-53. (in Chinese with English abstract)

[92]. Jacobs, J.L.; Ward, G.N. Effluent and nitrogen fertiliser effects on dry matter yield, nutritive characteristics, and mineral and nitrate content of turnips. *Aust. J. Agric. Res.* **2008**, *59*, 624–631.

[93]. Jadczak, D.; Grzeszczuk, M.; Kosecka, D. Quality characteristics and content of mineral compounds in fruit of some cultivars of sweet pepper (*capsicum annuum* L.). *J. Elem.* **2010**, *15*, 509–515.

[94]. Janes, B.E. The effect of irrigation, nitrogen level and season on the composition of cabbage. *Plant Physiol.* **1950**, *25*, 441-452.

[95]. Jayasinghe, G.Y. Composted sewage sludge as an alternative potting media for lettuce cultivation. *Commun. Soil Sci. Plant Anal.* **2012**.

[96]. Jurgiel-Malecka, G.; Gibczynska, M.; Nawrocka-Pezik, M. Comparison of chemical composition of selected cultivars of white, yellow and red onions. *Bulg. J. Agric. Sci.* **2015**, *21*, 736–741.

[97]. Kapoulas, N.; Koukounaras, A.; Ilić, Z.S. Nutritional quality of lettuce and onion as companion plants from organic and conventional production in north Greece. *Sci. Hortic.* **2017**, *219*, 310–318.

[98]. Kashem, M.A.; Kawai, S. Alleviation of cadmium phytotoxicity by magnesium in Japanese mustard spinach. *Soil Sci. Plant Nutr.* **2007**, *53*, 246–251.

[99]. Kawashima, L.M.; Valente Soares, L.M. Mineral profile of raw and cooked leafy vegetables consumed in Southern Brazil. *J. Food Compos. Anal.* **2003**, *16*, 605–611.

[100]. Kaymak, H.C.; Guvenc, I.; Gurol, A. Elemental analysis of different radish (*Raphanus Sativus* L.) cultivars by using wavelength-dispersive x-ray fluorescence spectrometry (WDXRF). *Bulg. J. Agric. Sci.* **2010**, *16*, 769–774.

[101]. Khan, N.K.; Watanabe, M.; Watanabe, Y. Effect of partial urea application on nutrient absorption by hydroponically grown spinach (*spinacia oleracea* L.). *Soil Sci. Plant Nutr.* **2000**, *46*, 199–208.

[102]. Kim, J.J.; John, K.M.M.; Hae-Kyung, M.; Jin, K.; Enkhtaivan, G.; Kim, D.H. Morphological and biochemical variation of Chinese cabbage (*Brassica rapa spp*. Pekinensis) cultivated using different agricultural practices. *J. Food Compos. Anal.* **2014**, *36*, 12–23.

[103]. Kiziloglu, F.M.; Turan, M.; Sahin, U.; Angin, I.; Anapali, O.; Okuroglu, M. Effects of wastewater irrigation on soil and cabbage-plant (*brassica olerecea* var. capitate cv. yalova-1) chemical properties. *J. Plant Nutr. Soil Sci.* **2007**, *170*, 166–172.

[104]. Kiziloglu, F.M.; Turan, M.; Sahin, U.; Kuslu, Y.; Dursun, A. Effects of untreated and treated wastewater irrigation on some chemical properties of cauliflower (*Brassica olerecea* L. var. botrytis) and red cabbage (*Brassica olerecea* L. var. rubra) grown on calcareous soil in Turkey. *Agric. Water Manag.* **2008**, *95*, 716–724.

[105]. Kopsell, D.E.; Kopsell, D.A.; Lefsrud, M.G.; Curran-Celentano, J. Variability in elemental accumulations among leafy brassica oleracea cultivars and selections . *J. Plant Nutr.* **2005**, *27*, 1813–1826.

[106]. Koudela, M.; Petříková, K. Nutrients content and yield in selected cultivars of leaf lettuce (*Lactuca sauva* L. var crispa). *Hortic. Sci.* **2008**, *35*, 99–106.

[107]. Kowalenko, C.G.; Ihnat, M. Effects of soil limestone and foliar manganese and zinc applications on field cauliflower growth and element contents. *Can. J. Soil Sci.* **2010**, *90*, 177–188.

[108]. Krelowska-Kulas, M. Determination of the level of certain trace elements in vegetables in differently contaminated regions. *Food. Nahrung* **1993**, *37*, 456–462.

[109]. Krzebietke, S. Response of butter lettuce (*Lactuca Sativa* L.) to different forms of nitrogen

fertilizers with chlorine and sulphates. *J. Elem.* **2008**, *13*, 581–588.
[110]. L. Bin; W. Xiao-zhong; G. Xi-lin; W.U. Gang; S. Yi-xiang; L. Long; G.E. Cheng-wen; C. Xin-ping. Nutrients absorption and distribution rule of pepper grown at autumn-winter season under mulched drop-irrigation system in greenhouse. *China. Veg.* **2017**, 50-57. (in Chinese with English abstract)
[111]. L. Liu; S. Cheng. Rapid determination of magnesium and zinc in turnip by flame atomic absorption spectrometry. *J. Liaoning. Normal. Univ.* **2004**, *27*, 327-329. (in Chinese with English abstract)
[112]. L. Yingli; G. Xiumin; F. Zheng; Di Bao. Analysis on nutrients component of 15 vegetables in Hebei. *Chin Agric. Sci. Bull.* **2007**, *23*, 98-100. (in Chinese with English abstract)
[113]. L.I. Zhong-yang; F. Xiang-yang; Q.I. Xue-bin; W.U. Hai-qing; DU Zhen-jie; Z. Zhi-juan; N. Yong-xing. Influences of reclaimed water irrigation on growth and quality of eggplant under different phosphate fertilizer treatments. *J. Irrig and Drain.* **2014**, *33*, 6-9. (in Chinese with English abstract)
[114]. Laczi, E.; Luca, E.; Dumitraş, A.; Hoaghia, A.; Boancă, P. Irrigation and fertilization management effect on chinese cabbage chemical composition. *Commun. Soil Sci. Plant Anal.* **2017**, *48*, 63–72.
[115]. Lamhamdi, M.; El Galiou, O.; Bakrim, A.; Nóvoa-Muñoz, J.C.; Arias-Estévez, M.; Aarab, A.; Lafont, R. Effect of lead stress on mineral content and growth of wheat (*Triticum aestivum*) and spinach (*Spinacia oleracea*) seedlings. *Saudi J. Biol. Sci.* **2013**, *20*, 29–36.
[116]. Lavilla, I.; Filgueiras, A. V.; Bendicho, C. Comparison of digestion methods for determination of trace and minor metals in plant samples. *J. Agric. Food Chem.* **1999**, *47*, 5072–5077.
[117]. Lee, J.; Lee, S. Correlations between soil physico-chemical properties and plant nutrient concentrations in bulb onion grown in paddy soil. *Sci. Hortic.* **2014**, *179*, 158–162.
[118]. Lee, J.; Son, D.; Hwang, S.; Min, B.; Kim, H.; Lee, S.; Kim, J.; Shim, S.; Boyhan, G.E. Effect of year, location, compost, and mixed oilseed cake on bulb and scale characteristic, nutrients and organic compounds in bulb and leaf, and storage quality in organic bulb onion. *J. Plant Nutr.* **2018**, *41*, 1636–1651.
[119]. Lee, T.J.; Luitel, B.P.; Kang, W.H. Growth and physiological response to manganese toxicity in Chinese cabbage (*Brassica rapa* L. ssp. campestris). *Hortic. Environ. Biotechnol.* **2011**, *52*, 252–258.
[120]. Lefsrud, M.G.; Kopsell, D.A.; Kopsell, D.E. Nitrogen levels influence biomass, elemental accumulations, and pigment concentrations in spinach. *J. Plant Nutr.* **2007**, *30*, 171–185.
[121]. Li, X.; Li, B.; Yang, Y. Effects of foliar selenite on the nutrient components of turnip (*Brassica rapa* var. rapa Linn.). *Front. Chem.* **2018**, *6*, 1–7.
[122]. Liopa-Tsakalidi, A.; Barouchas, P.E.; Xynias, I.N. Changes of elements concentration in onion set plants (*Allium cepa* L.) traditionally cultivated under different soil types in Greece. *Int. J. Agric. Res.* **2011**.
[123]. Lisk, D.J.; Furr, A.K.; Parkinson, T.F.; Gutenmann, W.H.; Pakkala, I.S. Elemental content of vegetables, grains, and forages field-grown on fly ash amended Soil. *J. Agric. Food Chem.* **1978**, *26*, 357–359.
[124]. Liu, M. Sen; Ko, M.H.; Li, H.C.; Tsai, S.J.; Lai, Y.M.; Chang, Y.M.; Wu, M.T.; Chen, L.F.O. Compositional and proteomic analyses of genetically modified broccoli (*Brassica oleracea* var. italica) harboring an agrobacterial gene. *Int. J. Mol. Sci.* **2014**, *15*, 15188–15209.
[125]. Lu, M.; Liu, D.; Shi, Z.; Gao, X.; Liang, Y.; Yao, Z.; Zhang, W.; Wang, X.; Chen, X. Nutritional quality and health risk of pepper fruit as affected by magnesium fertilization. *J. Sci. Food Agric.* **2020**.
[126]. Manas, D.; Soumya, G.; Kheyali, S. Effect of humic acid application on accumulation of mineral nutrition and pungency in garlic (*Allium sativum* L.). *Int. J. Biotechnol. Mol. Biol. Res.* **2014**, *5*, 7–12.
[127]. Mappaona, S.Y.; Kitou, M. Yield response of cabbage to several tropical green manure legumes incorporated into soil. *Soil Sci. Plant Nutr.* **1994**, *40*, 415–424.
[128]. Maria, A.; Santos, P.; Oliveira, A.C.; Souza, A.S.; Jesus, R.M. Determination and evaluation of the mineral composition of Chinese cabbage ( *Beta vulgaris* ). **2011**, 567–573.
[129]. Martínez, S.; Losada, P.; Franco, I.; Carballo, J. Protein, amino acid, ash and mineral contents in Brassica spp. grown in Northwest Spain. *Int. J. Food Sci. Technol.* **2011**, *46*, 146–153.
[130]. Matraszek, R.; Hawrylak-Nowak, B.; Chwil, S.; Chwil, M. Macronutrient balance of nickel-

stressed lettuce plants grown under different sulfur levels. *Commun. Soil Sci. Plant Anal.* **2017**, *48*, 665–682.
[131]. Meagy, M.J.; Eaton, T.E.; Barker, A. V. Assessment of mineral nutrient concentrations of lettuce in response to cultivar selection and fertilization in greenhouse production. *J. Plant Nutr.* **2016**, *39*, 1796–1808.
[132]. Meagy, M.J.; Eaton, T.E.; Barker, A. V; Bryson, G.M.; Eaton, T.E.; Barker, A. V; Bryson, G.M. Assessment of organic and conventional soil fertility practices and cultivar selection on mineral nutrient accumulation in field-grown lettuce. *J. Plant Nutr.* **2016**, *39*, 1936–1949.
[133]. Méndez, C.M.V.; Rodríguez, E.M.R.; Romero, C.D.; Matallana González, M.C.; Isasa, M.E.T. Comparison of the mineral and trace element concentrations between "gazpacho" and the vegetables used in its elaboration. *Int. J. Food Sci. Nutr.* **2008**, *59*, 660–670.
[134]. Michałojć, Z.; Buczkowska, H. Content of macroelements in eggplant fruits depending on varied potassium fertilization. *J. Elem.* **2009**, *14*, 111–118.
[135]. Miller-Ihli, N.J. Trace element determinations in foods and biological samples using inductively coupled plasma atomic emission spectrometry and flame atomic absorption spectrometry. *J. Agric. Food Chem.* **1996**, *44*, 2675–2679.
[136]. Minard, H.R.G. Effect of clove size, spacing, fertilisers, and lime on yield and nutrient content of garlic (*allium sativum*). *New Zeal. J. Exp. Agric.* **1978**, *6*, 139–143.
[137]. Mlček, J.; Valšíková, M.; Družbíková, H.; Ryant, P.; Juríková, T.; Sochor, J.; Borkovcová, M. The antioxidant capacity and macroelement content of several onion cultivars. *Turkish J. Agric. For.* **2015**, *39*, 999–1004.
[138]. Mohamed, A.E.; Rashed, M.N.; Mofty, A. Assessment of essential and toxic elements in some kinds of vegetables. *Ecotoxicol. Environ. Saf.* **2003**, *55*, 251–260.
[139]. Moreno-Caselles, J.; Prats, D.; Moral, R.; Perez-Murcia, M.D.; Perez-Espinosa, A.; Paredes, C.; León, V. Effects of linear alkylbenzene sulfonates (lass) in sewage sludge-amended soils on nutrient contents of broccoli plants. *Commun. Soil Sci. Plant Anal.* **2006**, *37*, 2605–2614.
[140]. Nascentes, C.C.; Korn, M.; Arruda, M.A.Z. A fast ultrasound-assisted extraction of Ca, Mg, Mn and Zn from vegetables. *Microchem. J.* **2001**, *69*, 37–43.
[141]. Ncayiyana, M.; Maboko, M.M.; Bertling, I. Alterations in yield, physicochemical components and mineral composition of onion following organic manure and inorganic nitrogen application. *Acta Agric. Scand. Sect. B Soil Plant Sci.* **2018**, *68*, 213–219.
[142]. Nishihara, E.; Inoue, M.; Kondo, K.; Takahashi, K.; Nakata, N. Spinach yield and nutritional quality affected by controlled soil water matric head. *Agric. Water Manag.* **2001**, *51*, 217–229.
[143]. Novozamsky, I.; Van Eck, R.; Houba, V.J.G.; Van Der Lee, J.J. Solubilization of plant tissue with nitric acid-hydrofluoric acid-hydrogen peroxide in a closed-system microwave digestor. *Commun. Soil Sci. Plant Anal.* **1996**, *27*, 867–875.
[144]. Nutrition, P. Effect of different magnesium doses on growth and yield. **2018**, *LXI*, 34–37.
[145]. Obregón-Cano, S.; Cartea, M.E.; Moreno, R.; De Haro-Bailón, A. Variation in glucosinolate and mineral content in Galician germplasm of *Brassica rapa* L. cultivated under Mediterranean conditions. *Acta Hortic.* **2018**, *1202*, 157–163.
[146]. Obregón-Cano, S.; Moreno-Rojas, R.; Jurado-Millán, A.M.; De Haro-Bailón, A. Anlisis del contenido en minerales en nabizas y grelos (*Brassica rapa* L. var. rapa) mediante reflectancia en el infrarrojo cercano. *CYTA - J. Food* **2016**, *14*, 359–367.
[147]. Obreza, T.A.; Vavrina, C.S. Production of Chinese cabbage in relation to nitrogen source, rate, and leaf nutrient concentration. *Commun. Soil Sci. Plant Anal.* **1993**, *24*, 1465–1479.
[148]. Ohtsuka, T.; Mo, W.; Uchida, M.; Sako, H.; Koizumi, H. Chapter 17 Slash-and-burn agriculture in a Japanese cedar (Cryptomeria japonica D. Don.) plantation: effects of fire on nutrients and soil emissions of carbon dioxide. *Elsevier Oceanogr. Ser.* **2007**, *73*, 395–416.
[149]. Ördög, A.; Poór, P.; Štajner, D.; Popović, B.; Bátori, Z.; Tari, I. Comparison of the mineral content of processed spice samples of sweet and hot paprika from the szeged region. *J. Elem.* **2018**, *23*, 521–530.
[150]. P. Shao; S. Li; S. Liu; P. Lan; X. Liao; R. Wang; Y. Zhang. Effects of B, Mo and Mg nutrition on yield and nutrients uptake of garlic giants. *North Hortic*. **2010**, 4-8. (in Chinese with English abstract)

[151]. Park, H.; Lee, S.; Jeong, H.; Cho, S.; Chun, H.; Back, O.; Kim, D.; Lillehoj, H.S. The nutrient composition of the herbicide-tolerant green pepper is equivalent to that of the conventional green pepper. *Nutr. Res.* **2006**, *26*, 546–548.
[152]. Pavlovich, L.B.; Strakhov, V.M. Effect of Humic Fertilizers from Brown Coal on the Mineral Composition of Vegetable Crops. *Solid Fuel Chem.* **2018**, *52*, 206–210.
[153]. Pennington, J.A.T.; Fisher, R.A. Food component profiles for fruit and vegetable subgroups. *J. Food Compos. Anal.* **2010**, *23*, 411–418.
[154]. Pennisi, G.; Orsini, F.; Blasioli, S.; Cellini, A.; Crepaldi, A.; Braschi, I.; Spinelli, F.; Nicola, S.; Fernandez, J.A.; Stanghellini, C.; et al. Resource use efficiency of indoor lettuce (*Lactuca sativa* L.) cultivation as affected by red:blue ratio provided by LED lighting. *Sci. Rep.* **2019**, *9*, 1–11, doi:10.1038/s41598-019-50783-z.
[155]. Perucka, I.; Olszówka, K. Accumulation of potassium, magnesium, calcium in fresh and cold stored leaves of lettuce (*Lactuca sativa* L.) after $CaCl_2$ foliar treatment before harvest. *J. Elemntology* **2011**.
[156]. Pinero, M.C.; Otálora, G.; Porras, M.E.; Sánchez-Guerrero, M.C.; Lorenzo, P.; Medrano, E.; Del Amor, F.M. The form in which nitrogen is supplied affects the polyamines, amino acids, and mineral composition of sweet pepper fruit under an elevated $CO_2$ concentration. *J. Agric. Food Chem.* **2017**, *65*, 711–717.
[157]. Pitura, K.; Michałojć, Z. Influence of nitrogen doses on the chemical composition and proportions of nutrients in selected vegetable species. *J. Elem.* **2015**, *20*, 667–676.
[158]. Pliszka, B.; Huszcza-Ciołkowska, G.; Mieleszko, E.; Wróblewska-Wierzbicka, B.; Januszewicz, E. Using two methods for plant material preparation in order to determine the content of bioelements in red cabbage (*Brassica Oleracea* L. var. Capitata L. F. Rubra). *J. Elem.* **2011**, *16*, 455–465.
[159]. Pokluda, R. Nutritional quality of Chinese cabbage from integrated culture. *Hortic. Sci.* **2008**, *35*, 145–150.
[160]. Põldma, P.; Moor, U.; Tõnutare, T.; Herodes, K.; Rebane, R. Selenium treatment under field conditions affects mineral nutrition, yield and antioxidant properties of bulb onion (*Allium cepa* L.). *Acta Sci. Pol. Hortorum Cultus.* **2013**, *12*, 167–181.
[161]. Põldma, P.; Tõnutare, T.; Viitak, A.; Luik, A.; Moor, U. Effect of selenium treatment on mineral nutrition, bulb size, and antioxidant properties of garlic (*Allium sativum* L.). *J. Agric. Food Chem.* **2011**, *59*, 5498–5503.
[162]. Pongrac, P.; McNicol, J.W.; Lilly, A.; Thompson, J.A.; Wright, G.; Hillier, S.; White, P.J. Mineral element composition of cabbage as affected by soil type and phosphorus and zinc fertilisation. *Plant Soil* **2019**, *434*, 151–165.
[163]. Prasad, M.; Spiers, M. Comparative Study of Ashing Techniques for the Digestion of Horticultural Plant Samples. *J. Agric. Food Chem.* **1978**, *26*, 824–827.
[164]. Przybysz, A.; Wrochna, M.; Gawrońska, H.; Małecka-Przybysz, M.; Pietrzyk, S.; Gawroński, S.W. Effect of manganese on yield and quality of hydroponically grown lettuce. *J. Elem.* **2017**, *22*, 315–327.
[165]. Przybysz, A.; Wrochna, M.; Małecka-Przybysz, M.; Gawrońska, H.; Gawroński, S.W. The effects of Mg enrichment of vegetable sprouts on Mg concentration, yield and ROS generation. *J. Sci. Food Agric.* **2016**, *96*, 3469–3476.
[166]. Przybysz, A.; Wrochna, M.; Małecka-Przybysz, M.; Gawrońska, H.; Gawroński, S.W. Enrichment of some leafy vegetables with magnesium. *J. Elem.* **2016**, *21*, 797–809.
[167]. Przygocka-Cyna, K.; Grzebisz, W.; Łukowiak, R.; Biber, M. Biomass ash and biogas digestate bio-fertilizers as a source of nutrients for light acid soil – an exhaustion lettuce test. *J. Elem.* **2018**, *23*, 413–428.
[168]. Publishing, C. Milk production responses to turnips fed to dairy cows in mid lactation. *Aust. J. Exp. Agric.* **2000**, *40*, 681–696.
[169]. Q. Zhang; L. Liu; H. Wang. Determination of calcium and magnesium in eggplant by flame atomic absorption spectrometry. *Mod. Instrum*. **2005**, 19-21. (in Chinese with English abstract)
[170]. Qin, J.; Shi, A.; Mou, B.; Grusak, M.A.; Weng, Y.; Ravelombola, W.; Bhattarai, G.; Dong, L.; Yang, W. Genetic diversity and association mapping of mineral element concentrations in spinach

leaves. *BMC Genomics* **2017**, *18*, 1–14.
[171]. Radziemska, M.; Vaverková, M.D.; Adamcová, D.; Brtnický, M.; Mazur, Z. Valorization of Fish Waste Compost as a Fertilizer for Agricultural Use. *Waste Biomass Valorization* **2019**, *10*, 2537–2545.
[172]. Rani, V.; Khetarpaul, N.; Sharma, S.K. Mineral Profile of Tomato, Cabbage and Brinjal Irrigated With Canal Water and Sodic Water Treated With Gypsum and Farm Yard Manure. *Indian J. Agric. Res.* **2015**, *47*, 226–231.
[173]. Ribeiro, H.M.; Romero, A.M.; Pereira, H.; Borges, P.; Cabral, F.; Vasconcelos, E. Evaluation of a compost obtained from forestry wastes and solid phase of pig slurry as a substrate for seedlings production. *Bioresour. Technol.* **2007**, *98*, 3294–3297.
[174]. Roosta, H.R. Effects of foliar spray of K on mint, radish, parsley and coriander plants in aquaponic system. *J. Plant Nutr.* **2014**, *37*, 2236–2254.
[175]. Roosta, H.R. Interaction between water alkalinity and nutrient solution ph on the vegetative growth, chlorophyll fluorescence and leaf magnesium, iron, manganese, and zinc concentrations in lettuce. *J. Plant Nutr.* **2011**, *34*, 717–731.
[176]. Rubio, C.; Hardisson, A.; Martín, R.E.; Báez, A.; Martín, M.M.; Álvarez, R. Mineral composition of the red and green pepper (*Capsicum annuum*) from Tenerife Island. *Eur. Food Res. Technol.* **2002**, *214*, 501–504.
[177]. Ruiz, J.M.; Moreno, D.A.; Romero, L. Pyruvate kinase activity as an indicator of the level of $K^+$, $Mg^{2+}$, and $Ca^{2+}$ in leaves and fruits of the cucumber: The role of potassium fertilization. *J. Agric. Food Chem.* **1999**, *47*, 845–849.
[178]. Ryu, H.D.; Lim, C.S.; Kang, M.K.; Lee, S.I. Evaluation of struvite obtained from semiconductor wastewater as a fertilizer in cultivating Chinese cabbage. *J. Hazard. Mater.* **2012**, *221–222*, 248–255.
[179]. S. Guohan; Y.L.L. Effect of magnesium concentration on growth and development and magnesium absorption of cabbage (*Brassica oleracea* L. var. capitata). *Shandong Agric Sci*. **1998**, 10-14. (in Chinese with English abstract)
[180]. Sahin, U.; Kuslu, Y.; Kiziloglu, F.M. Response of cucumbers to different irrigation regimes applied through drip-irrigation system. *J. Anim. Plant Sci.* **2015**, *25*, 198–205.
[181]. Sajid, M.; Butt, M.S.; Shehzad, A.; Tanweer, S. Chemical and mineral analysis of garlic: a golden herb. *Pakistan J. Food Sci.* **2014**, *24*, 108–110.
[182]. Samuolienė, G.; Brazaitytė, A.; Viršilė, A.; Miliauskienė, J.; Vaštakaitė-Kairienė, V.; Duchovskis, P. Nutrient levels in brassicaceae microgreens increase under tailored light-emitting diode spectra. *Front. Plant Sci.* **2019**, *10*, 1–9.
[183]. Sánchez-Torres, P.; Raigón, M.D.; Gammoudi, N.; Gisbert, C. Effects of grafting combinations on the nutritional composition of pepper fruit. *Fruits* **2016**, *71*, 249–256.
[184]. Schnepf, M.; Driskell, J. Sensory attributes and nutrient retention in selected vegetables prepared by conventional and microwave methods. *J. Food Qual.* **1994**.
[185]. Sengul, M.; Yildiz, H.; Ercisli, S.; Yildirim, E.; Turan, M.; Ozdemir, O.; Sener, D. Some phytochemical characteristics of turnip (*Brassica rapa* var. rapa L.) roots. **2011**, *23*. ISBN 5857939585.
[186]. Seo, M.W.; Yang, D.S.; Kays, S.J.; Kim, J.H.; Woo, J.H.; Park, K.W. Effects of nutrient solution electrical conductivity and sulfur, magnesium, and phosphorus concentration on sesquiterpene lactones in hydroponically grown lettuce (*Lactuca sativa* L.). *Sci. Hortic.* **2009**.
[187]. Shimomachi, T.; Kawahara, Y.; Kobashigawa, C.; Omoda, E.; Hamabe, K.; Tamaya, K. Effect of residual salinity on spinach growth and nutrient contents in polder soil. *Acta Hortic.* **2008**, *797*, 419–424.
[188]. Siddique, S. Enhancement of growth and productivity of cucumber (*Cucumis sativus*) through foliar application of calcium and magnesium. *Pure Appl. Biol.* **2017**, *6*, 402–411.
[189]. Sikorska-Zimny, K.M.; Wędzisz, A.; Rogowska, M. Spinosad decay dynamics and mineral content of some elements in chosen vegetables. *J. Elem.* **2017**, *22*, 737–746.
[190]. Šindelářová, K.; Száková, J.; Tremlová, J.; Mestek, O.; Praus, L.; Kaňa, A.; Najmanová, J.; Tlustoš, P. The response of broccoli (*Brassica oleracea* convar. italica) varieties on foliar application of selenium: uptake, translocation, and speciation. *Food Addit. Contam. - Part A Chem. Anal. Control. Expo. Risk Assess.* **2015**, *32*, 2027–2038.
[191]. Smith, C.B.; Demchak, K.T.; Ferretti, P.A. Fertilizer placement effects on growth responses and

nutrient uptake of sweet corn, snapbeans, tomatoes and cabbage. *Commun. Soil Sci. Plant Anal.* **1990**, *21*, 107–123.
[192]. Smoleń, S.; Sady, W. Effect of plant biostimulation with Pentakeep V fertilizer and nitrogen fertilization on the content of macro- and micronutrients in Spinach. *J. Elem.* **2010**, *15*, 343–354.
[193]. Smoleń, S.; Sady, W. Influence of soil application of iodine and sucrose on mineral composition of spinach plants. *Acta Sci. Pol. Hortorum Cultus* **2011**, *10*, 3–13.
[194]. Sousa, A.A.T.C.; Figueiredo, C.C. Sewage sludge biochar: Effects on soil fertility and growth of radish. *Biol. Agric. Hortic.* **2016**, *32*, 127–138.
[195]. Stagnari, F.; Di Bitetto, V.; Pisante, M. Effects of N fertilizers and rates on yield, safety and nutrients in processing spinach genotypes. *Sci. Hortic.* **2007**, *114*, 225–233.
[196]. Stanojkovic-sebic, A.; Dinić, Z.; Postic, D.; Savic, D.; Ilicic, R.; Josic, D.; Pivic, R. Levels of macro and trace elements in vegetable crops as influenced by Fresenius. *Environ. Bull.* **2017**, *26*, 1017–1025.
[197]. Sziklai, I.L.; Ördögh, M.; Molnár, E.; Szabó, E. Distribution of trace and minor elements in hungarian spice paprika plants. *J. Radioanal. Nucl. Chem. Artic.* **1988**, *122*, 233–238.
[198]. Tadesse, T.; Nichols, M.A.; Fisher, K.J. Nutrient conductivity effects on sweet pepper plants grown using a nutrient film technique: 1. Yield and fruit quality. *New Zeal. J. Crop Hortic. Sci.* **1999**, *27*, 229–237.
[199]. Tadesse, T.; Nichols, M.A.; Hewett, E.W.; Fisher, K.J. Relative humidity around the fruit influences the mineral composition and incidence of blossom-end rot in sweet pepper fruit. *J. Hortic. Sci. Biotechnol.* **2001**, *76*, 9–16.
[200]. Toom, M.; Talgre, L.; Mäe, A.; Tamm, S.; Narits, L.; Edesi, L.; Haljak, M.; Lauringson, E. Selecting winter cover crop species for northern climatic conditions. *Biol. Agric. Hortic.* **2019**, *35*, 263–274.
[201]. Turan, M.; Kordali, S.; Zengin, H.; Dursun, A.; Sezen, Y. Macro and micro mineral content of some wild edible leaves consumed in Eastern Anatolia. *Acta Agric. Scand. Sect. B Soil Plant Sci.* **2003**, *53*, 129–137.
[202]. Udoessien, E.I.; Ifon, E.T. Chemical studies on the unripe and ripe fruits of Dennettia tripatela (pepper fruit). *Food Chem.* **1984**, *13*, 257–263.
[203]. Urbano, V.R.; Mendonça, T.G.; Bastos, R.G.; Souza, C.F. Effects of treated wastewater irrigation on soil properties and lettuce yield. *Agric. Water Manag.* **2017**, *181*, 108–115.
[204]. Uusiku, N.P.; Oelofse, A.; Duodu, K.G.; Bester, M.J.; Faber, M. Nutritional value of leafy vegetables of sub-Saharan Africa and their potential contribution to human health: A review. *J. Food Compos. Anal.* **2010**, *23*, 499–509.
[205]. Van Jaarsveld, P.; Faber, M.; van Heerden, I.; Wenhold, F.; Jansen van Rensburg, W.; van Averbeke, W. Nutrient content of eight African leafy vegetables and their potential contribution to dietary reference intakes. *J. Food Compos. Anal.* **2014**, *33*, 77–84.
[206]. W. Hui-min; J. Xiao-yong; L. Ying-chao; C. Zhu-jun; C. Jing-yang; Z. Jian-bin. Influence of foliar spraying magnesium on uptake and distribution of nutrients in magnesium deficient tomato grown under greenhouse. *Agric. Res. Arid. Area*. **2017**, *35*, 226-231. (in Chinese with English abstract)
[207]. W. Zhang; L. Xie; Q. Ji; C. Fu; S. Pan; G. Liu. Response of Capsicum annuum to nitrogen, phosphorus, potassium and magnesium. *J. Agric*. **2011**, *1*, 37-40. (in Chinese with English abstract)
[208]. Wang, L.; Guo, S.; Wang, Y.; Yi, D.; Wang, J. Poultry biogas slurry can partially substitute for mineral fertilizers in hydroponic lettuce production. *Environ. Sci. Pollut. Res.* **2019**, *26*, 659–671.
[209]. Warman, P.R. Soil fertility, yield and nutrient contents of vegetable crops after 12 years of compost or fertilizer amendments. *Biol. Agric. Hortic.* **2005**, *23*, 85–96.
[210]. Wen, G.; Bates, T.E.; Voroney, R.P.; Winter, J.P.; Schellenberg, M.P. Influence of application of sewage sludges, and sludge and manure composts on plant Ca and Mg concentration and soil extractability in field experiments. *Nutr. Cycl. Agroecosystems* **1999**, *55*, 51–61.
[211]. Wen, G.; Huang, L.; Zhang, X.; Hu, Z. Uptake of nutrients and heavy metals in struvite recovered from a mixed wastewater of human urine and municipal sewage by two vegetables in calcareous soil. *Environ. Technol. Innov.* **2019**, *15*, 100384.
[212]. Wolnik, K.A.; Fricke, F.L.; Capar, S.G.; Meyer, M.W.; Satzger, R.D.; Bonnin, E.; Gaston, C.M. Elements in major raw agricultural crops in the united states. 3. cadmium, lead, and eleven other

elements in carrots, field corn, onions, rice, spinach, and tomatoes. *J. Agric. Food Chem.* **1985**, *33*, 807–811.
[213]. Wong, J.W.C.; Li, G.X.; Wong, M.H. The growth of Brassica chinensis in heavy-metal-contaminated sewage sludge compost from Hong Kong. *Bioresour. Technol.* **1996**, *58*, 309–313.
[214]. Wu, J.; Yuan, Y.X.; Zhang, X.W.; Zhao, J.; Song, X.; Li, Y.; Li, X.; Sun, R.; Koornneef, M.; Aarts, M.G.M.; et al. Mapping QTLs for mineral accumulation and shoot dry biomass under different Zn nutritional conditions in Chinese cabbage (*Brassica rapa* L. ssp. pekinensis). *Plant Soil* **2008**, *310*, 25–40.
[215]. X. Sun. Effects of magnesium, manganese, boron and molybdenum on nutrient content of lettuce. *Qinghai Sci.Tech*. **2005**, 27-29. (in Chinese with English abstract)
[216]. X. Wu; S. Zhang; H. Wu; L. Chang; J. Mu. Absorption characteristics of calcium, magnesium and sulphur of overwintering spinach in eastern Hebei province. *J. Hebei Vocat.-Tech. Teach. Coll*. **2003**, *17*, 6-10. (in Chinese with English abstract)
[217]. X. Xiaoyu; Z. Bing. Effect of Mg concentration on the Mg content, yield and quality of cucumber. *J. Agric Mech. Res*. **2012**, *34*, 145-148. (in Chinese with English abstract)
[218]. X.U. Hui-qin; Y.U. Bao-xia. Determin the microelement magnesium in different kinds of turnips by atomic absorption spectrophotometry. *Hebei Chem. Eng. Ind*. **2010**, *33*, 66-67. (in Chinese with English abstract)
[219]. Xiao, Z.; Codling, E.E.; Luo, Y.; Nou, X.; Lester, G.E.; Wang, Q. Microgreens of Brassicaceae: Mineral composition and content of 30 varieties. *J. Food Compos. Anal.* **2016**, *49*, 87–93.
[220]. Xu, L.L.; Fan, Z.Y.; Dong, Y.J.; Kong, J.; Liu, S.; Hou, J.; Bai, X.Y. Effects of exogenous NO supplied with different approaches on cadmium toxicity in lettuce seedlings. *Plant Biosyst.* **2015**, *149*, 270–279.
[221]. Y. Liang. Effects of magnesium fertilizer and its distribution under drip irrigation. *J. Anhui. Agric. Sci*. **2014**, *42*, 5066-5070. (in Chinese with English abstract)
[222]. Ye, X.H.; Zhao, Z.L.; Zhou, Q.; Zhao, H.B.; Guo, C.M.; Shi, L.L.; Guo, J.W. Malabsorption of mineral nutrients and effects of foliar fertilization on continuously cropped *Capsicum annuum* L. var. annuum. *Pakistan J. Bot.* **2014**, *46*, 1781–1788.
[223]. Yildirimand, E.; Turan, M. Growth, yield and mineral content of broccoli intercropped with lettuce. *J. Anim. Plant Sci.* **2013**, *23*, 919–922.
[224]. Yoldas, F.; Ceylan, S.; Yagmur, B.; Mordogan, N. Effects of nitrogen fertilizer on yield quality and nutrient content in broccoli effects of nitrogen fertilizer on yield quality. **2008**, *4167*.
[225]. Z. Guo; Y. Wang; Y. Zhang; X. Zhu; Z. Shen; W. Xu. Component analysis of 8 elements including potassium, calcium and magnesium in cucumber, winter melon, balsam pear, towel gourd and watermelon. *China Cucurbits Veg*. **2019**, *32*, 25-29. (in Chinese with English abstract)
[226]. Z. Li; Z. Huang. Determination of Mg, Ca, Zn, Cu, Mn and Fe in garlic by microwave digestion-atomic absorption spectrometry. *Stud. Trace Elem. Health*. **2006**, 40-42. (in Chinese with English abstract)
[227]. Z. Yang. Effects of calcium and magnesium fertilizer on yield, quality and nutrient absorption of tomato. *Soils Fert.* **1994**, 14-18. (in Chinese with English abstract)
[228]. Z. Yang; W. Tao. Effects of different concentrations of calcium and magnesium nutrient solution on growth and development of tomato and its relationship with element absorption. *Chin. J. Soil Sci*. **1994**, 190-193. (in Chinese with English abstract)
[229]. Zahradník, A.; Petříková, K. Effect of alternative organic fertilizers on the nutritional value and yield of head cabbage. *Hortic. Sci.* **2007**, *34*, 65–71.
[230]. Zandvakili, O.R.; Barker, A. V.; Hashemi, M.; Etemadi, F.; Autio, W.R. Comparisons of commercial organic and chemical fertilizer solutions on growth and composition of lettuce. *J. Plant Nutr.* **2019**, *42*, 990–1000.
[231]. Zandvakili, O.R.; Ebrahimi, E.; Hashemi, M.; Barker, A. V.; Akbari, P. The potential of green manure mixtures to provide nutrients to a subsequent lettuce crop. *Commun. Soil Sci. Plant Anal.* **2017**, *48*, 2246–2255.
[232]. Zemanová, V.; Břendová, K.; Pavlíková, D.; Kubátová, P.; Tlustoš, P. Effect of biochar application on the content of nutrients (Ca, Fe, K, Mg, Na, P) and amino acids in subsequently

growing spinach and mustard. *Plant, Soil Environ.* **2017**, *63*, 322–327.
[233]. Zhao, Y.; Hu, C.; Wang, X.; Qing, X.; Wang, P.; Zhang, Y.; Zhang, X.; Zhao, X. Selenium alleviated chromium stress in Chinese cabbage (*Brassica campestris* L. ssp. Pekinensis) by regulating root morphology and metal element uptake. *Ecotoxicol. Environ. Saf.* **2019**, *173*, 314–321.
[234]. Zheljazkov, V.D.; Horgan, T.E.; Astatkie, T.; Fratesi, D.; Mischke, C.C. Study on shrimp waste water and vermicompost as a nutrient source for bell peppers. *HortScience* **2011**, *46*, 1493–1496.
[235]. Żurawik, A.; Jadczak, D.; Żurawik, P. Content of macro- and microelements in the yield of garlic chives (Allium tuberosum Rottler ex Spreng.) according to the plant age. *J. Elemntology.* **2013**, *18*, 3-15.
